# Supplementary material for: The interactome of intact mitochondria by cross-linking mass spectrometry provides evidence for coexisting respiratory supercomplexes
Source: Mol Cell Proteomics. 2017 Dec 8;17(2):216–32. doi: 10.1074/mcp.RA117.000470 (PMC5795388; doi:10.1074/mcp.RA117.000470)
Supplement: Supplemental Data [file supp_RA117.000470_133922_0_supp_23978_fzffwf.zip › spectra_annotation/mito_spectra_annotation/364-1-6-1-7-1.pdf]

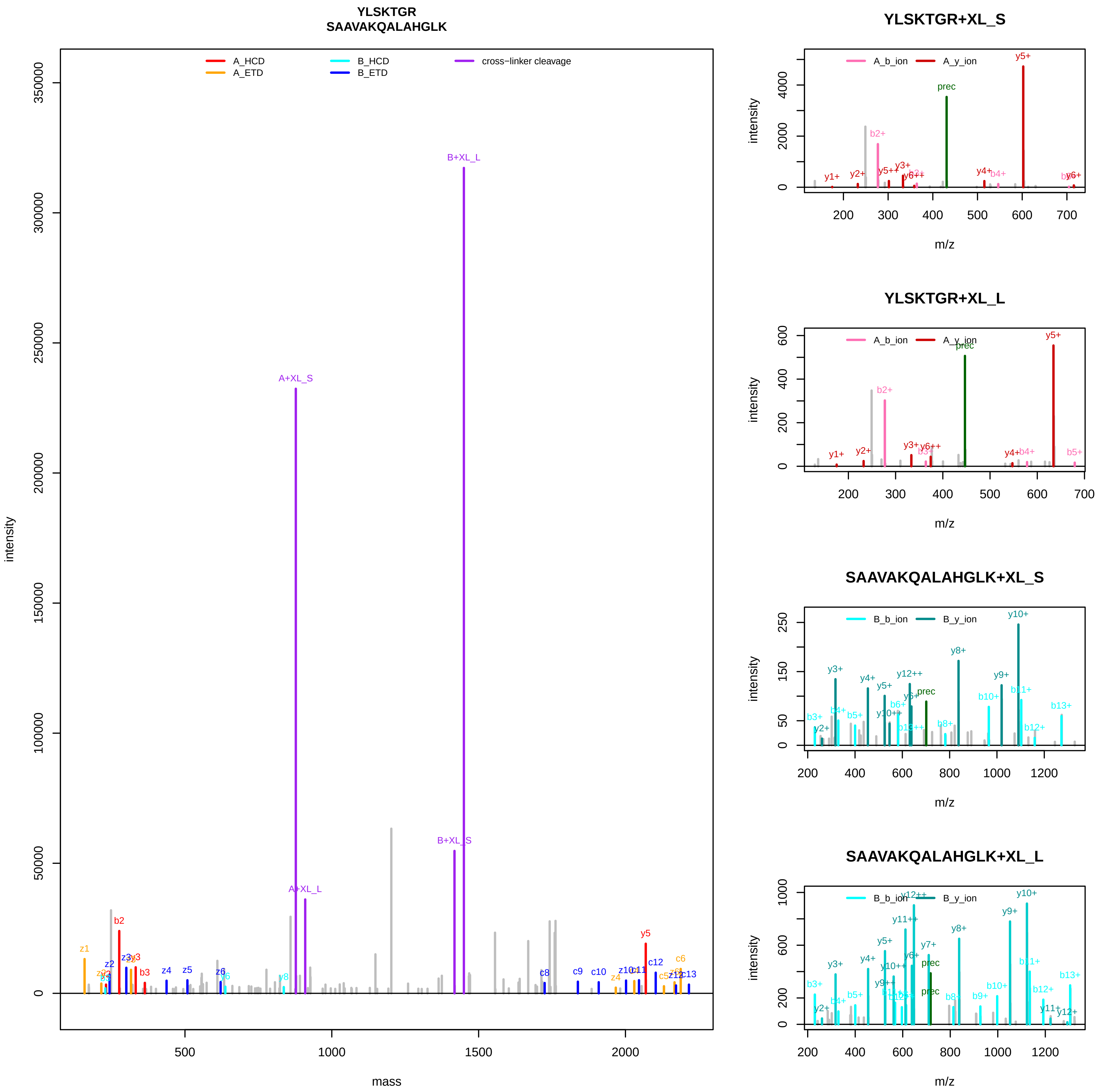

YLSKTGR
SAAVAKQALAHGLK
A_HCD
A_ETD
B_HCD
B_ETD
cross−linker cleavage
B+XL_L
A+XL_S
B+XL_S
A+XL_L
intensity
mass
0
50000
100000
150000
200000
250000
300000
350000
500
1000
1500
2000
z1
b2
b3
y3
z3
z2
z4
z5
z6
y6
y8
c8
c9
c10
z10
c11
c12
c13
y5
c5
c6
YLSKTGR+XL_S
YLSKTGR+XL_L
SAAVAKQALAHGLK+XL_S
SAAVAKQALAHGLK+XL_L
A_b_ion
A_y_ion
B_b_ion
B_y_ion
prec
y5+
y4+
b4+
y1+
y2+
b2+
y3+
y6++
b5+
y10+
y8+
y9+
b10+
b11+
b12+
b13+
y12++
m/z
intensity
200
300
400
500
600
700
800
1000
1200
